# Noun–noun combination: Meaningfulness ratings and lexical statistics for 2,160 word pairs

**DOI:** 10.3758/s13428-012-0256-3

**Published:** 2012-10-06

**Authors:** William W. Graves, Jeffrey R. Binder, Mark S. Seidenberg

**Affiliations:** 1 Department of Psychology, Rutgers University, Smith Hall Room 337, 101 Warren Street, Newark, NJ 07102 USA; 2 Department of Neurology, Medical College of Wisconsin, Milwaukee, WI USA; 3 Department of Psychology, University of Wisconsin – Madison, Madison, WI USA

**Keywords:** Conceptual combination, Lexical, Semantics, Ratings, Concepts

## Abstract

The combining of individual concepts to form an emergent concept is a fundamental aspect of language, yet much less is known about it than about processing isolated words or sentences. To facilitate research on conceptual combination, we provide meaningfulness ratings for a large set of (2,160) noun–noun pairs. Half of these pairs (1,080) are reversed versions of the other half (e.g., ski jacket and jacket ski), to facilitate the comparison of successful and unsuccessful conceptual combination independently of constituent lexical items. The computer code used for obtaining these ratings through a Web interface is provided. To further enhance the usefulness of this resource, ancillary measures obtained from other sources are also provided for each pair. These measures include associate production norms, contextual relatedness in terms of latent semantic analysis distance, total number of letters, phrase-level usage frequency, and word-level usage frequency summed across the words in each pair. Results of correlation and regression analyses are also provided for a quantitative description of the stimulus set. A subset of these stimuli was used to identify neural correlates of successful conceptual combination Graves, Binder, Desai, Conant, & Seidenberg, (NeuroImage 53:638–646, 2010). The stimuli can be used in other research and also provide benchmark data for evaluating the effectiveness of computational algorithms for predicting meaningfulness of noun–noun pairs.

**Electronic supplementary material:**

The online version of this article (doi:10.3758/s13428-012-0256-3) contains supplementary material, which is available to authorized users.

## Introduction

Much work has been performed in experimental psychology and cognitive neuroscience examining mechanisms of single word- and sentence-level processing (Balota, Yap, & Cortese, 2006; Binder & Price, 2001; Démonet, Thierry, & Cardebat, 2005; Gernsbacher & Kaschak, 2003; Graves, Binder, Seidenberg, & Desai, 2012; Kaan & Swaab, 2002; Martin, 2003; Pulvermüller, 1999; Schlaggar & McCandliss, 2007). Considerably less is known about the processing of intermediate-level linguistic units, such as the noun–noun phrases that are common in English and other languages. Such expressions require combinatorial semantic processes; the meaning of the phrase is typically more than a simple conjunction of the meanings of the parts. The aim of this study was to identify a large set of noun–noun pairs that, once rated in forward (meaningful) and reversed orders, could be used to examine various aspects of combinatorial processing (for the first use of such stimuli, see Seidenberg, Waters, Sanders, & Langer, 1984). The combining of a pair of words, each representing a distinct concept, into a phrase that derives its meaning from both words is often referred to as *conceptual combination*. A growing body of work has examined the cognitive mechanisms underlying how concepts are combined (for a review, see Murphy, 2002) and, more recently, their neural correlates (Baron & Osherson, 2011; Bemis & Pylkkänen, 2011; Graves, Binder, Desai, Conant, & Seidenberg, 2010). The meaningfulness of the stimuli is typically determined from subjective investigator judgments (e.g., Gagné & Shoben, 1997), corpus counts (Gagné & Spalding, 2009), or ratings of a limited set of stimuli (Wisniewski & Love, 1998; Wisniewski & Murphy, 2005). Given the continuing interest in and importance of conceptual combination, our aim was to overcome these limitations by providing meaningfulness ratings for a large set of concept pairs.

As an example of the utility of this stimulus set, 400 of the phrases that were rated as very meaningful in one direction (e.g., lake house) and not meaningful when reversed (house lake) were used in a functional magnetic resonance imaging (fMRI) study of the neural correlates of successful conceptual combination (Graves et al., 2010). The main contrast of interest compared the neural responses to meaningful phrases with the neural responses to phrases containing the same pair of words but in reversed order. This revealed a primarily right-hemisphere set of brain regions, including the angular gyrus and dorsomedial prefrontal cortex. Primarily left-lateralized versions of these areas are consistently implicated across functional brain-imaging studies of lexical semantic processing (Binder, Desai, Graves, & Conant, 2009). A different set of left-lateralized regions, including the posterior middle temporal and parahippocampal gyri, was identified by an analysis of the summed frequency of the words in each phrase. This analysis was performed to highlight areas engaged in lexical-level processing, which were also among those consistently identified in previous imaging studies of lexical semantic processing (Binder et al., 2009). By revealing brain regions involved in conceptual combination as distinct from lexical-level processing, the previous study (Graves et al., 2010) illustrated one of many possible uses of the ratings presented here. Other possibilities include comparing meaningfulness ratings to noun–noun pairs across different levels of phrase-level usage frequency or comparing the human ratings of meaningfulness with those based on meaningfulness metrics derived from computational algorithms such as latent semantic analysis (LSA: Landauer, McNamara, Dennis, & Kintsch, 2007). The relevant values are provided with the noun–noun pairs presented here.

Before presenting the methodological details, we note that all nouns making up these phrases were rated as relatively high in imageability, a dimension closely related to concreteness, in previous studies (min = 6, max = 6.8 on a 1 to 7 scale; Bird, Franklin, & Howard, 2001; Clark & Paivio, 2004; Cortese & Fugett, 2004; Gilhooly & Logie, 1980; Paivio, Yuille, & Madigan, 1968; Toglia & Battig, 1978). One of the main applications envisioned for these ratings is in studies of conceptual combination comparing meaningful compounds with less meaningful or nonmeaningful compounds with the same constituent words. To this end, we excluded noun–noun pairs consisting of abstract constituents. Such phrases are likely to be judged as intermediate in meaningfulness, making meaningful conceptual combination less clear than for concrete noun pairs.

The usefulness of this full set of ratings is also expected to be enhanced by providing the actual computer code used to obtain them (see supplemental material). The procedure required minimal experimenter involvement, and the participants could complete the ratings using any computer with Internet access. Once submitted, participant ratings were appended directly to a master data file on a remote laboratory server. This code can be modified to fit any number of new scenarios, allowing control over parameters such as what participants see and how the data are handled.

## Method

Stimulus selection began by choosing the 500 most concrete words available from a composite imageability database compiled from six imageability rating studies (Bird et al., 2001; Clark & Paivio, 2004; Cortese & Fugett, 2004; Gilhooly & Logie, 1980; Paivio et al., 1968; Toglia & Battig, 1978). These words were then checked for noun status using the CELEX lexical database (Baayen, Piepenbrock, & Gulikers, 1995). Words were retained only if their noun frequency was greater than their frequency in other parts of speech. All possible pairwise combinations of these nouns were generated, resulting in *n*(*n −* 1), or 249,500, potential phrases. A large database of human-generated text from Internet-based USENET groups (Shaoul & Westbury, 2007) was searched electronically for the occurrence of these phrases. Phrases appearing at least once in this corpus and in only one direction (i.e., “noun1 noun2,” but not “noun2 noun1”) were retained, resulting in a total of 1,351 potentially meaningful phrases. These were read over by one of the investigators, who removed potentially problematic items such as those that were apparently nonsensical, might have interchangeable word order, or were taboo phrases, resulting in a final set of 1,080 phrases for rating. Note that some of the words combined to form phrases more often than did others. There were 321 unique words in the noun1 position and 298 unique words in the noun2 position.

The full set was split into five equal subsets of 216 phrases each, with a reversed-phrases counterpart generated for each set. For a given participant, one of the five forward-phrase sets and one of the five reversed-phrase sets were concatenated together, their order randomized, and presented for rating. The combination of forward and reversed sets was constrained such that no participant saw forward and reversed versions of the same phrases.

Participants (*N* = 150) were recruited from the psychology student participant pool at the University of Wisconsin–Madison and provided informed consent. For participant tracking and to award course credit, each participant was given a World Wide Web address to which to point a browser and a random tracking number that ended in a number between 1 and 5. This determined which of the five sets the participant was given to rate. The order of presentation of phrases within each set was rerandomized for each participant. Upon entering the Web address, participants were given instructions for making their ratings using the full range of a 5-point scale (values 0–4) as follows:

Please read each phrase, then judge how meaningful it is as a single concept, using a scale from 0 to 4 as follows: If the phrase makes no sense, the appropriate rating is 0. If the phrase makes some sense, the appropriate rating is 2. If the phrase makes complete sense, the appropriate rating is a 4. Please indicate your response by clicking on the button to the left of the number. Please consider the full range of the scale when making your ratings.

The following examples were given as anchor points: the goat sky, 0 (*makes no sense*), the fox mask, 2 ( *makes some sense*), the computer programmer, 4 ( *makes complete sense*). After entering the tracking number and clicking “submit,” the ratings page was displayed. Upon completing the ratings and clicking “submit,” the data were appended to a master file that resided on a laboratory file server.

Ratings were checked for outlier status on a per-participant basis. Since each participant saw only one of five lists, mean ratings for each item were calculated within list and checked for correlation with each participant’s ratings. Data from any participant giving ratings with a correlation more than 2 standard deviations (*SD*s) from the mean were eliminated, as were data from 1 participant who failed to complete a majority of the ratings. This resulted in elimination of data from 1 to 2 participants per list, or 8 out of 150 participants. Each phrase was rated by a minimum of 26 participants. A full list of the noun–noun pairs, along with all ancillary data, is provided as an Excel file in the supplemental material.

### Sources of ancillary measures

Association ratings were obtained from two independent sources (Kiss, Armstrong, Milroy, & Piper, 1973; Nelson, McEvoy, & Schreiber, 1998). These contained results from studies of word associate production in which participants were given a probe word and were asked to produce the first associated word that came to mind. The associate produced may or may not be semantically related to the probe. For example, “car” was produced as an associate of “police” by over 10% of participants, although these words belong to different semantic categories (artifacts vs. persons). The associate production norms are also directional. For example, “police” was never produced as an associate for the probe word “car.”

In contrast to participant-based ratings such as meaningfulness and association, all other ancillary measures were based on either text corpora (for LSA, word frequency and phrase frequency) or surface properties (number of letters). The LSA measure reported here was calculated as the cosine distance between vectors representing two words in high-dimensional semantic space, with higher numbers reflecting ostensibly greater semantic or contextual relatedness (Landauer & Dumais, 1997). This was performed using the “pairwise comparison” tool available from the LSA Web site (http://lsa.colorado.edu). A word frequency measure was obtained for each phrase by log_10_-transforming the frequency of each word in the phrase as it appears in CELEX (Baayen et al., 1995) and summing across the two words. This method was chosen to be sensitive to the frequency of the individual words in each phrase. In contrast, phrase-level frequency was obtained by log_10_-transforming the number of times each noun–noun combination occurred in a Google-derived count of the contents of the World Wide Web as of 2006 (Brants & Franz, 2006). Finally, number of letters was simply a count of the total number of letters in each phrase.

## Results

### Overall distributions and descriptive statistics

**Fig. 1:**
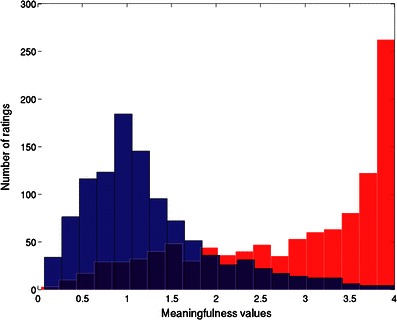
Distribution of meaningfulness values grouped by forward (red) or reversed (blue) order, each divided into 20 discrete bins

As was expected, the meaningfulness ratings for the forward (meaningful) and reversed (less meaningful) pairs were strongly and reliably different, with forward phrases having a mean meaningfulness rating of 2.83 and reversed order phrases having a mean rating of 1.23 (*t* = 40.31, *p* < .001; Table 1). As can be seen in the histogram overlays in Fig. 1, the distributions of meaningfulness values for the two categories of phrases overlap but are largely distinct. The distinction can be quantified by counting the number of occurrences of each value. The modal mean value for meaningfulness of forward phrases (averaged across participants) is 4.00, occurring 87 times. The modal mean value for meaningfulness of reversed phrases is 0.96, occurring 30 times. Ratings for the forward phrases have a leftward skew (i.e., the mean of 2.83 is smaller than the median of 3.15) due to the values clustering at the top of the scale. Hence, this set offers the options of selecting forward or reversed phrases from overlapping or distinct regions of the distributions.

**Table 1:** Summary statistics for the entire set of noun-noun pairs and separated according to whether they were expected to be meaningful (forward order) or not meaningful (reversed order)

Factor	Mean	Median	*SD*
All pairs			
Meaningfulness	2.03	1.69	1.22
Association	0.02	0.00	0.06
LSA	0.19	0.15	0.16
Number of letters	9.87	10	2.23
Phrase frequency (log)	3.14	3.23	1.27
Summed word frequency (log)	5.76	5.81	0.88
Forward			
Meaningfulness	2.83	3.15	1.07
Association	0.02	0.00	0.07
Phrase frequency (log)	3.81	3.90	0.98
Reversed			
Meaningfulness	1.23	1.07	0.73
Association	0.01	0.00	0.05
Phrase frequency (log)	2.46	2.62	1.16

*SD* = standard deviation.

Differences between forward and reversed order phrases are also reflected in the phrase-level log frequency count (forward = 3.81 and reversed = 2.46; see Table 1). The 1.35 difference on the log scale corresponds to relatively large differences in raw frequency. For example, the mean of the untransformed (raw) frequency counts for forward phrases is 39,562 and for reversed is 2,706, indicating a large difference between the two phrase types. Both log-transformed and untransformed values are available for all items in the supplemental material.

### Correlations between measures

In Table 2, correlations were examined between meaningfulness ratings, the standard deviation of meaningfulness ratings, and all of the variables in Table 1. The rationale for including the standard deviation of meaningfulness was to get a sense of the degree of consistency across ratings. Each item has a mean meaningfulness rating value and a corresponding standard deviation. Correlations with association are based on the subset of 2,144 items for which association values were available. Most of the pairings were only modestly correlated but still significant at *p* < .05, due to the large number of observations. The largest correlation (*r* = .61, *p* < .0001) is between meaningfulness and the phrase-level Google-derived frequency estimate (Brants & Franz, 2006). Of note, the correlation between meaningfulness and the raw (i.e., not log-transformed) phrase-level frequency was much lower (*r* = .23), supporting the common practice of log-transforming frequency values. The only correlation with meaningfulness that did not reach significance was for the sum of the individual word frequencies, as derived from CELEX (Baayen et al., 1995). This lack of correlation suggests that the participants based their meaningfulness ratings, as instructed, on phrase-level judgments, rather than information specific to individual words.

**Table 2:** Pairwise correlations (Pearson *r*-values) among all variables of interest

	Meaningfulness	*SD*	Association	LSA	Length	Phrase freq
SD	**−.34**					
Association	**.16**	**−.12**				
LSA	**.19**	**−.12**	**.35**			
Length	.06^*^	−.02	−.02	.01		
Phrase freq	**.61**	**−.19**	**.19**	**.23**	−.03	
Sum word freq	−.01	.05^†^	**−.09**	**−.16**	.01	**.23**

Values are based on the subset of items for which association measures were available (2,144 of 2,160). *SD*, standard deviation; LSA, latent semantic analysis; frq, frequency. Entries in bold were significant at *p* < .0001.

^*^ *p* < .01

^**†**^ *p* < .05

Total number of letters (letter length) for each pair showed a modest but reliable correlation with meaningfulness ratings (*r* = .06, *p* < .01). This correlation was surprising, since number of letters is not considered to be a variable related to meaning (Balota, Cortese, Sergent-Marshall, Spieler, & Yap, 2004; New, Ferrand, Pallier, & Brysbaert, 2006; Weekes, 1997). Unlike other meaning-correlated variables, however, letter length did not correlate with the standard deviation for meaningfulness (*r* = −.02, *p* > .05). This suggests that letter length bears a relationship to meaningfulness that is different from that of variables such as whole-phrase frequency or distance in semantic space that are more straightforwardly related to semantics.

**Fig. 2:**
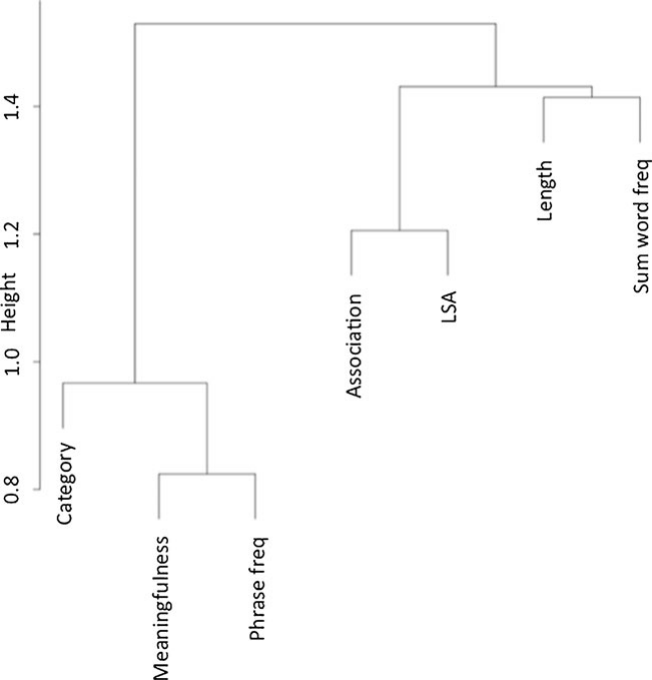
Hierarchical cluster analysis based on squared Spearman rank correlation distances showing the relationships between the factors in Table 2

To further characterize the relationship of the letter length predictor to the other predictors, we performed a cluster analysis in which the square of the Spearman rank correlation was used as the basis of the distance metric for hierarchical clustering. This analysis was implemented in the R statistical package using the “hclust” function, as described in Baayen (2008). As is shown in Fig. 2, the first split is between a cluster of three variables—category (forward or reversed), meaningfulness rating, and phrase frequency—and the remaining four variables—association, LSA, summed word frequency, and letter length. The latter cluster was split into one cluster containing association and LSA and another containing summed word frequency and letter length. Thus, rather than relating to phrase-level meaning, letter length seems to be more related to the lexical constituents of the phrases.

### Regression analyses using ancillary measures

In addition to individual pairwise correlations, multiple linear regression analyses were performed to examine the degree to which the ancillary variables uniquely contribute to predicting the meaningfulness ratings. This was done for the subset of 2,144 (out of 2,160) phrases for which association measures were available. The influence on meaningfulness ratings of the following five factors was examined: association, LSA distance, letter length, phrase frequency, and summed word frequency (Table 3). These variables together accounted for 41% of the variance in meaningfulness. Only association and LSA distance failed to significantly predict meaningfulness. Phrase frequency was the strongest predictor (*ß* = .65, *p* < .0001), followed by summed word frequency (*ß* = −.16, *p* < .0001), and letter length (*ß* = .08, *p* < .0001). The direction of the summed word frequency result is perhaps surprising in that having lower frequency constituent words predicted higher meaningfulness ratings. This result however, along with the letter length result, should be interpreted with caution. Although they were statistically reliable (at *p* < .0001), having a large number of observations can inflate the chances of finding results that, while statistically reliable, show effects small enough to be considered negligible. Indeed, the *R* ^2^ values for summed word frequency (.108) and letter length (.002) suggest that together they explain only about 11% of the variance in meaningfulness ratings.

**Table 3:** Results of regression analyses for the subset of 2,144 noun–noun phrases for which association measures were available

Predictor	Meaningfulness		*SD*	
	Beta	*p*	Beta	*p*
Association	.02	.32	−.07	.00
LSA	.01	.64	−.04	.09
Length	.08	.00	−.03	.23
Phrase freq	.65	.00	−.18	.00
Sum word freq	−.16	.00	.08	.00

Values indicate the ability of five explanatory variables to predict average meaningfulness ratings (columns 2 and 3) or to predict the standard deviation of the ratings (two rightmost columns). These values represent standardized regression weights (beta weights) and corresponding *p*-values from tests of significance

An additional regression analysis was performed that was identical to the previous one, except that phrase category (forward = 1, reversed = ─ − 1) was added to the analysis to test whether or not the other factors predicted continuous meaningfulness ratings beyond that explained by a priori phrase category (Table 4). This model accounted for 55% of the variance in meaningfulness. As was expected, category status was the most predictive of meaningfulness ratings (*ß* = .46, *p* < .0001). This was followed by phrase frequency (*ß* = .37, *p* < .0001), with letter length being the least predictive, although still reliable (*ß* = .07, *p* < .0001). Adding category to the regression model revealed an effect of LSA distance (*ß* = .09, *p* < .0001) that was not present in the previous model. This suggests that, if left unmodeled, the effect of phrase category overwhelms the effect of LSA distance. LSA distance may also be particularly sensitive to within-category variance in meaningfulness ratings.

**Table 4:** Results of regression analyses reported as in Table 3, but with phrase category (forward or reversed) added as a predictor variable

Predictor	Meaningfulness		*SD*	
	Beta	*p*	Beta	*p*
Category	.46	.00	−.05	.04
Association	.02	.26	−.07	.00
LSA	.09	.00	−.05	.04
Length	.07	.00	−.02	.26
Phrase freq	.37	.00	−.15	.00
Sum word freq	−.08	.00	.07	.00

LSA, latent semantic analysis; freq, frequency

## Discussion

Here, we have provided a resource to aid in the investigation of conceptual combination. This resource consists of both human ratings and relevant ancillary data for a large set of noun–noun combinations. Computational tools are also provided in the form of computer scripts to automate the process of additional data collection. The manipulation performed here of presenting noun–noun phrases in either forward (lake house) or reversed (house lake) order succeeded in that it led to a bimodal distribution of meaningfulness values (Fig. 1). This characteristic of the stimulus set should be useful in enabling further investigation of meaningful and less meaningful, or optimal and suboptimal, conceptual combination.

Covariance among variables was also examined, with two goals in mind. One was to characterize the stimulus set as fully as possible. The other was to begin exploring the data set for potentially interesting or unexpected relationships that might lead to further fruitful study. For example, it seems that after including presentation order (forward or reversed) in the regression analysis of meaningfulness ratings (Table 4), LSA distance was able to uniquely account for variance in meaningfulness ratings. This finding might be predicted on the basis of claims that the LSA measure is related to text-based meaning (Landauer & Dumais, 1997). A more surprising finding from this analysis was that meaningfulness ratings were also predicted by overall letter length of the phrases. This association could not obviously be attributed to an indirect influence through a third variable, since letter length was not correlated with any of the other predictor variables. Results of the cluster analysis shown in Fig. 2 suggest that length is more strongly related to summed word frequency, a lexical variable based on phrase constituents, than to phrase-level semantic variables such as meaningfulness. Further exploration of the possible relationship between meaningfulness and letter length is only one of many potentially fruitful uses of the resource provided here for the study of noun–noun phrases.

For investigators preparing to use these phrases, it may be worth noting that several of the constituent words are repeated a number of times across phrases, as described above in the Method section. This offers the option of choosing a subset of the phrases containing unique words. Alternatively, one could take advantage of the fact that, although several of the constituent words repeat, each phrase in the corpus is unique. This would, for example, allow investigators to choose phrases that elicit word-level repetition priming (or possibly repetition suppression in the case of functional neuroimaging; Grill-Spector, Henson, & Martin, 2006) without phrase-level priming.

While this stimulus set is, to our knowledge, the largest of its kind, Wisniewski and Murphy (2005) collected similar ratings in their study reexamining evidence for the competition among relations in nominals model of conceptual combination (Gagné & Shoben, 1997). Wisniewski and Murphy collected ratings on familiarity (participants indicated phrases they had “heard or seen before”), plausibility (on a scale of 1 to 7 with 1 being *very weird* and 7 being *very plausible*), and frequency of phrase occurrence based on number of hits from the Google Internet search engine (similar to the phrase-frequency measure used in the present study). Analyses revealed phrase frequency and familiarity to be highly correlated at either *r* = .60 or .50, depending on the set of phrases being considered.^1^ These correlations are very similar to the correlation found in the present study between phrase frequency and meaningfulness (.61). This convergence of findings points to the replicability of the Wisniewski and Murphy results in a larger data set and the external validity of the present results. We hope that this data set will serve as a helpful resource for future studies of conceptual combination.

## Electronic supplementary material

Below is the link to the electronic supplementary material.

ESM 1 (XLSX 340 kb)

ESM 2 (ZIP 21.5 kb)

## Footnotes

1 Although Wisniewski and Murphy (2005) did not report the correlation between phrase frequency and plausibility, it is likely to have been similar to familiarity, because plausibility and familiarity were correlated at .90 and .92, again depending on the set of phrases being considered.
